# Recent Advances in the Molecular Mechanisms Underlying Pyroptosis in Sepsis

**DOI:** 10.1155/2018/5823823

**Published:** 2018-03-07

**Authors:** Yu-Lei Gao, Jian-Hua Zhai, Yan-Fen Chai

**Affiliations:** Department of Emergency Medicine, Tianjin Medical University General Hospital, Tianjin 300052, China

## Abstract

Sepsis is recognized as a life-threatening organ dysfunctional disease that is caused by dysregulated host responses to infection. Up to now, sepsis still remains a dominant cause of multiple organ dysfunction syndrome (MODS) and death among severe condition patients. Pyroptosis, originally named after the Greek words “*pyro*” and “*ptosis*” in 2001, has been defined as a specific programmed cell death characterized by release of inflammatory cytokines. During sepsis, pyroptosis is required for defense against bacterial infection because appropriate pyroptosis can minimize tissue damage. Even so, pyroptosis when overactivated can result in septic shock, MODS, or increased risk of secondary infection. Proteolytic cleavage of gasdermin D (GSDMD) by caspase-1, caspase-4, caspase-5, and caspase-11 is an essential step for the execution of pyroptosis in activated innate immune cells and endothelial cells stimulated by cytosolic lipopolysaccharide (LPS). Cleaved GSDMD also triggers NACHT, LRR, and PYD domain-containing protein (NLRP) 3-mediated activation of caspase-1 via an intrinsic pathway, while the precise mechanism underlying GSDMD-induced NLRP 3 activation remains unclear. Hence, this study provides an overview of the recent advances in the molecular mechanisms underlying pyroptosis in sepsis.

## 1. Introduction

Sepsis is defined as a life-threatening pathological alteration to severe condition patients hospitalized in intensive care units. During the past decades, researches have determined a significant increase in the morbidity of sepsis [1–3]. Despite that a better understanding for the pathophysiology of sepsis has improved the outcome of disease, in-hospital deaths from sepsis still remarkably rise [1, 4]. Besides, the life quality of survivors is evidently impaired, characterized by more physical dysfunction, dementia and/or cognitive defects, higher readmission rates, and even increased long-term mortality [5–7]. In China, both the morbidity and mortality of sepsis gradually increase with age, irrespective of gender, although male patients exhibit slightly higher age-adjusted morbidity and mortality. The morbidity of sepsis also shows seasonal fluctuations, especially reaching the peak in cold winter. Epidemiological studies demonstrated that the mortality of sepsis reached up to 20.6%, implying that the standardized mortality rate was 79/100,000 every year [8, 9].

During sepsis, immunoinflammatory response when moderately regulated can equip the organism with effective defense against pathogenic microorganism and consequently mild tissue damage, whereas a severe immunoinflammatory dysfunction, especially an excessive proinflammatory dysfunction or immunosuppression, commonly leads to organ dysfunction or secondary infection [2, 3]. Although sepsis has been extensively studied over the past decades, the mechanism underlying the significant pathophysiological alterations remains unclear. Accumulating evidence suggests that inflammasome, an intracellular multiprotein complex, is involved in the pathogenesis of sepsis [10, 11]. The inflammasome triggered pyroptosis in a caspase-1-dependent manner [12, 13]. In sepsis, pyroptosis ruptures the plasma membrane, which results in releases of abundant inflammatory factors [10–15]. An obvious deficiency in the number of immunocytes, especially gastrointestinal epithelial cells, dendritic cells (DCs), lymphocytes, and even thymocytes, was observed in both septic animals and patients [16, 17]. Without any effective treatments, septic patients would undergo primary hyperinflammatory responses followed by immunosuppression, that is, immunoparalysis [2]. Recently, extensive studies have been focused on elucidating the molecular mechanisms underlying immunoparalysis and developing novel strategies to effectively regulate immune homeostasis during sepsis, such as activation of regulatory T cells (Tregs) and apoptotic depletion of immunocytes [7, 18–20].

**Figure 1:**
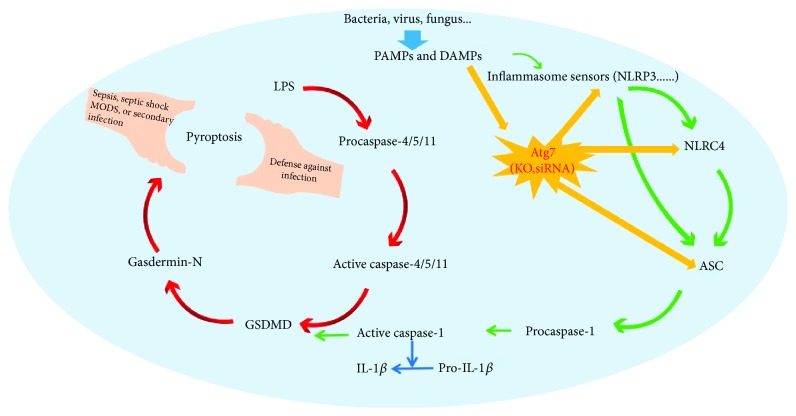
Pyroptosis and sepsis. Pore-forming activity of GSDMD determines pyroptotic cell death. Direct binding of LPS to the protein procaspase-11 causes the protein dimerization to become active caspase-11. In human cells, caspase-4 and caspase-5 performed the function of mouse caspase-11. These caspases cleaved the protein GSDMD. The cytotoxic N-terminal fragment of GSDMD was then released and targets phospholipids on the host cell membrane. The NLRP 3 pathway is triggered by LPS molecules in the cytoplasm of infected cells. The inflammasome sensors recruit procaspase-1 monomers through the adaptor protein ASC and activate the caspase by dimerization. Caspase-1 can also initiate pyroptosis by cleaving gasdermin D although other pyroptosis-inducing caspase-1 substrates may exist. During sepsis, pyroptosis is required for defense against bacterial infection because appropriate pyroptosis can minimize tissue damage. Even so, pyroptosis when overactivated can result in septic shock, MODS, or increased risk of secondary infection.

Pyroptosis is an inflammatory form of programmed cell death. In general, pyroptosis protects multicellular host organisms against invasive pathogenic bacteria and microbial infections; however, it also causes sepsis and septic shock when overactivated (Figure 1) [15, 21–23]. In the 20th century, the discovery of Toll-like receptor- (TLR-) 4 was a key landmark for understanding innate- and specific-immune responses in infectious diseases, especially in sepsis. Unfortunately, these efforts were not adequate in clinical trials due to a lack of efficacy [24, 25]. In 2013, two critical studies explained this discrepancy by demonstrating a noncanonical inflammasome activation pathway independent of TLR-4 but dependent on detection of cytoplasmic LPS through caspase-11 activation [26, 27]. More and more studies suggested that caspase-11 is directly activated by LPS, wherein LPS is bound to the caspase activation and recruitment domain (CARD) [28–31]. Caspase-11 is considered crucial for organisms to defend against intracellular Gram-negative bacteria, which further established this noncanonical pathway as the primary defense against intracellular bacterial infection. For a long time, caspase-1 activation is identified as the primary step for pyroptosis. Caspase-1, which belongs to the inflammasome-related caspase family, cleaves and facilitates the maturation of prointerleukin- (IL-) 1*β* into IL-1*β* [32, 33]. Damage-associated molecular pattern molecules (DAMPs) are released by pyroptotic cells, which contain the matured form of IL-1*β*. These DAMPs then recruit innate- and specific-immune cells to the infectious lesions. Inflammatory caspases (caspase-1, caspase-4, caspase-5, and caspase-11) are critical for immune defenses [34–36]. Caspase-1 is activated by ligands of typical inflammasomes, while other inflammatory caspases directly recognize LPS. Both events force cells to undergo pyroptosis [32, 37, 38]. In spite that the central effects of pyroptosis in immunity and endotoxic shock have been determined, the mechanism underlying pyroptosis induced by inflammatory caspases is still ambiguous. Pyroptosis is mechanistically distinct from other types of cell death.

## 2. GSDMD Was the Pivotal Substrate of Pyroptosis in Sepsis

The GSDM family contains four paralogs in vertebrates, named as GSDMA, GSDMB, GSDMC, and GSDMD. All GSDM proteins share two predicted domains with a variable linker region, which are distinct from any other known domain structures [26, 27, 39]. In 2015, two studies simultaneously identified GSDMD as the pivotal and direct target of caspase-11 in the noncanonical inflammasomes when stimulated by cytosolic LPS. Kayagaki et al. found that GSDMD was necessary for the formation of caspase-11-mediated pyroptosis and IL-1*β* secretion. A forward genetic screen established the correlation between *Gsdmd* and intracellular LPS responses in mice with ethyl-*N*-nitrosourea- (ENU-) induced dominant negative mutation. Macrophages isolated from gene-targeting-generated *Gsdmd^−/−^* mice also escaped from pyroptosis and produced less IL-1*β* under the stimulation of cytoplasmic LPS or G^−^ bacteria (including *Salmonella typhimurium*, *Escherichia coli*, *Burkholderia thailandensis*, and *Shigella flexneri*) [39]. In addition, *Gsdmd^−/−^* mice were remarkably resistant to LPS, as evidenced by the higher lethal dose. Cleavage products of GSDMD by caspase-11, especially the amino-terminal fragment, determine pyroptosis and NLRP 3-triggered caspase-1 activation [40, 41]. By contrast, using genome-wide clustered regularly interspaced palindromic repeat- (CRISPR-) Cas-9 nuclease screens of caspase-11 and caspase-1 mediated pyroptosis in mouse bone marrow macrophages (BMDMs) for guide RNAs that prevented LPS-caused death. Shi et al. demonstrated that pyroptosis was obviously abrogated by GSDMD deficiency in cells stimulated with cytosolic LPS and typical inflammasome ligands. Further, IL-1*β* secretion was also receded in *Gsdmd^−/−^* cells even in the presence of caspase-1 [42]. Caspase-1 and caspase-4/5/11 specifically recognized and then cleaved the peptide bonds between the amino- and carboxy-terminal domains in GSDMD, which was the core event in pyroptosis [35, 36, 42].

Gasdermin-N domain was released from intramolecular repressive domain to trigger intrinsic pyroptosis [42]. However, other family members of gasdermin exhibited autoinhibition without being cleaved by inflammatory caspases; genetic mutations in the C-terminal domain of Gsdma3, gain-of-function mutations, blocked the autoinhibition, facilitating gasdermin-N domain-mediated pyroptosis. Thus, several negative effects were caused, among which alopecia and skin defects were the most important [40, 43]. These findings not only provided novel insights into inflammasome-dependent immunity/diseases but also clearly elucidated the difference between pyroptosis and necroptosis. Both research groups found that *Gsdmd^−/−^* BMDMs were resistant to LPS-induced pyroptosis and expressed decreased matured IL-1*β* upon LPS stimulation. Further, they also demonstrated that both *Gsdmd^−/−^* and *Casp11^−/−^* mice survived from LPS with lethal doses, authenticating the role of GSDMD in pyroptosis-triggered lethal sepsis and septic shock [40, 42].

## 3. NLRP 3 Inflammasome-Enlarged Pyroptosis in Sepsis

The inflammasome which promotes the maturation and secretion of proinflammatory cytokines (e.g., IL-1*β* and IL-18) is a molecular platform in the immunologic system [10–13]. The NLRP family members are critical components of inflammasomes, which function through interaction with apoptosis-associated speck-like protein containing CARD (ASC) and subsequent recruit of the precursor form of caspase-1 [10, 44]. There are two component pathways that typically activate NLRP 3 inflammasome, that is, the TLR4-ligand pathway and ATP-dependent purinergic receptor pathway [10, 11, 45]. Unlike other inflammasomes, NLRP 3 inflammasome is a highly impressionable factor to both pathogen-associated molecular patterns (PAMPs) and DAMPs. After being stimulated by diverse PAMPs and DAMPs, NLRP 3 recruits ASC and caspase-1, leading to activation of caspase-1, maturation and secretion of IL-1*β* and IL-18, and initiation of pyroptosis [10, 46–48].

The role of NLRP 3 inflammasome has been established in various immune and inflammatory diseases [44, 49]. The activation of NLRP 3 inflammasome is important for protecting cells from pathogenic microbes; however, excessive activated NLRP 3 inflammasome may induce hyperinflammation and cause tissue damage, organ failure, and even death [10, 44, 49]. Wu and colleagues established a classic septic model in rats by cecal ligation and puncture (CLP) and studied the role of NLRP3 in sepsis by injecting rats with *Nlrp 3* short hairpin RNA plasmids (*Nlrp 3* shRNA). They found that *Nlrp 3* knockdown attenuated hyperbileacidaemia through restoration of the abundance of hepatocyte transporters and suppression of the production of hepatic cytokines, neutrophil infiltration, and macrophage pyroptosis during sepsis [49]. NLRP 3 could be a promising molecular target for sepsis treatment. Activation of NLRP 3 inflammasome commonly results in neutrophil infiltration in organs, such as the liver and kidney. The resident macrophages, such as Kupffer cells (KCs) in the liver, undergo pyroptosis in an inflammasome-dependent manner during sepsis, contributing to organ dysfunction [49, 50]. The expression of NLRP 3 was significantly increased in the liver during sepsis [49]. Therefore, NLRP 3 may be a promising target for sepsis-induced multiple organ dysfunction.

NLRP 3 inflammasome can also be activated by danger signals released from stressed cells or pathogens, such as viruses and bacteria. The dysregulation of NLRP 3 inflammasome activation also causes and aggravates several diseases, especially sepsis [10, 46–49]. Therefore, investigating the strategies to limit NLRP 3 inflammasome activation is necessary for human health. Recent studies have indicated that mitochondrial dysfunction was closely correlated with NLRP 3 inflammasome activation and pyroptosis during sepsis [51–53]. Sepsis causes mitochondrial damage by an unknown mechanism, and the damaged mitochondria release mitochondrial ROS (mtROS) identified as danger signals [54]. Damaged mitochondria in cells treated with NLRP 3 inflammasome activators release enhanced danger signals, especially mtROS and mtDNA [51, 52, 55]. Another study consolidated this concept that increased mtROS and mtDNA were detected in plasma samples of septic patients [54]. The evidence shows that both the NLRP 3 inflammasome and damaged mitochondria contribute to sepsis.

With the pore formation of GSDMD in the presence of caspase-11 when stimulated by LPS, the NLRP 3 inflammasome is activated [40–42]. After stimulated by intracellular LPS, the maturation and secretion of IL-1*β* requires caspase-1, NLRP 3, and ASC, suggesting that NLRP 3 inflammasome activation is engaged by caspase-11 in the presence of LPS stimulation. By contrast, these factors are not required for induction of pyroptosis, implying that the NLRP 3 inflammasome activation occurs after GSDMD cleavage [46, 47, 56]. Indeed, the activation of caspase-1 and the process of IL-1*β* halted in *Gsdmd^−/−^* BMDMs stimulated with intracellular LPS, suggesting that GSDMD mediates NLRP 3 inflammasome activation [40]. It is conceivable that NLRP 3 inflammasome is directly activated by GSDMD-N; however, such a mechanism is difficult to reconcile with the rapid membrane localization and pore formation of GSDMD-N. It is likely that potassium efflux after the pore formation of GSDMD-N triggers the noncanonical activation pathway of NLRP 3 inflammasome.

## 4. Autophagy-Related Protein 7 and Pyroptosis in Sepsis

Autophagy-related (*Atg*) genes are indispensable regulators of autophagy by controlling the induction and formation of autophagophore under stress or poor nutrient conditions [57, 58]. Atg 7 is homologous to the ubiquitin-activating enzyme E1 (Uba1) in the two ubiquitin-like conjugation systems, which is essential to these conjugation systems and indispensable for both selective and nonselective autophagy inductions in innate immunity [15, 57, 59]. Recently, by *i.p.* challenge with *P. aeruginosa*, Pu and her colleagues showed that Atg7 was involved in inflammasome activation and pyroptosis in the septic model. *Atg7^fl/fl^* mice showed impaired pathogen clearance, decreased survival, and widespread dissemination of bacteria into blood and organs, such as the lung. Loss of *Atg7* increased production of IL-1*β* and pyroptosis, which was consistent with enhanced inflammasome activation [15]. These results provided new insights into the function of Atg7 in host defense during bacterial sepsis.

Previous studies showed that autophagy played specific roles in degrading intracellular pathogens in a cell-autonomous manner and orchestrating systemic immunoinflammatory responses by regulating the immune system and inflammation [57]. Silence of *Atg* genes caused heavier bacterial burdens of macrophages, and deletion of *Atg* genes in plants and *Drosophila* increased viral replication, mortality, and more severe pathological phenotypes [60, 61]. Knockout of *Atg7* increased tissue injury and inflammatory responses, especially maturation and secretion of IL-1*β* and activation of pyroptosis [15]. Atg7 linked the immune cross-talk between autophagy and pyroptosis pathways in bacterial infection, particularly in *P. aeruginosa*-induced septic progression.

The NLRP 3 inflammasome is activated during *P. aeruginosa*-induced sepsis, while silencing the NLRP 3 inflammasome in macrophages impedes the clearance of *P. aeruginosa*, which might be attributed to the weakened autophagy flux [61]. Noteworthily, IL-1*β* stimulation diminished the lethality of macrophages to *P. aeruginosa*, which, however, was restored by *Atg7* knockdown [15]. Cathepsin B (CTSB) induced by Atg7 improved the impairment of glucose-stimulated insulin secretion, which was markedly abolished by NLRP 3 deficiency in pancreatic INS-1 (823/13) cells. Therefore, NLRP 3 inflammasome could be identified as an autophagic component [62–64]. Combined together, CTSB arouses Atg7-induced NLRP 3-dependent proinflammatory responses. Pu and her colleagues demonstrated that multiple types of inflammasomes with diverse functions can be activated during sepsis [15]. The interrelationship between autophagy and pyroptosis pathways is complicated and can be affected by various elements, including pathogens, actuation duration, host defense capability, and animal models [15, 64]. Hence, *Atg7* deletion may contribute to enhanced pyroptosis, and the molecular mechanism underlying bacterial infection and sepsis progression should be further studied. Knockout of *Atg16L1* led to increased amounts of inflammatory cytokines (IL-1*β* and IL-18) in the presence of LPS stimulation. Other researches showed that disordered autophagy was associated with the activation of proatherogenic inflammasomes, which promoted atherosclerosis in part through inflammasome hyperactivation [65, 66]. Likewise, in Pu and her colleagues' study, they observed overproduction of IL-1*β* and intensified pyroptosis in septic *atg7^fl/fl^* mice. Particularly, transfection of flagellin into *atg7^fl/fl^* macrophages also led to inflammasome hyperactivation, suggesting that autophagy could regulate the host immune response to flagellin via an unknown mechanism [15]. This study primarily focused on the role of immune cells in inflammasome activation, which can be a hot topic of future researches on sepsis.

## 5. Conclusions

Studies from the last several years have propelled our understanding of pyroptosis and its signaling pathways. There is now clear evidence to conclude that pyroptosis protects multicellular host organisms against invaded pathogenic bacteria and microbial infections; however, it also causes sepsis and septic shock when overactivated. Biochemical and structural studies have identified new components and regulators of the pyroptosis in sepsis. DAMPs are released by pyroptotic cells and then recruit innate- and specific-immune cells to the infectious lesions. Binding between a single entity of ligand and an inflammasome sensor can induce a cascade of oligomerization events, culminating in pyroptosis, which define the physiologic outcomes in the host. Therefore, further studies unraveling the molecular basis of pyroptosis activation at a structural and biochemical level would inform translational studies.

## Abbreviations

MODS: Multiple organ dysfunction syndrome
GSDMD: Gasdermin D
LPS: Lipopolysaccharide
NLRP 3: NACHT, LRR, and PYD domain-containing protein 3
DCs: Dendritic cells
Tregs: Regulatory T cells
TLR: Toll-like receptor
CARD: The caspase activation and recruitment domain
IL: Interleukin
DAMPs: Damage-associated molecular pattern molecules
ENU: Ethyl-*N*-nitrosourea
CRISPR: Clustered regularly interspaced palindromic repeat
BMDMs: Bone marrow macrophages
ASC: Apoptosis-associated speck-like protein containing CARD
PAMPs: Pathogen-associated molecular patterns
CLP: Cecal ligation and puncture
MtROS: Mitochondria release mitochondrial reactive ROS
Atg: Autophagy-related
CTSB: Cathepsin B.
